# Supplementary material for: Genomic and environmental determinants of circular single-stranded DNA production in engineered E. coli
Source: bioRxiv. 2026 Oct 29:2026.10.28.761454. Preprint. [Version 1] doi: 10.64898/2026.10.28.761454 (PMC13733975; doi:10.64898/2026.10.28.761454)
Supplement: Supplementary file 1 [file media-1.pdf]

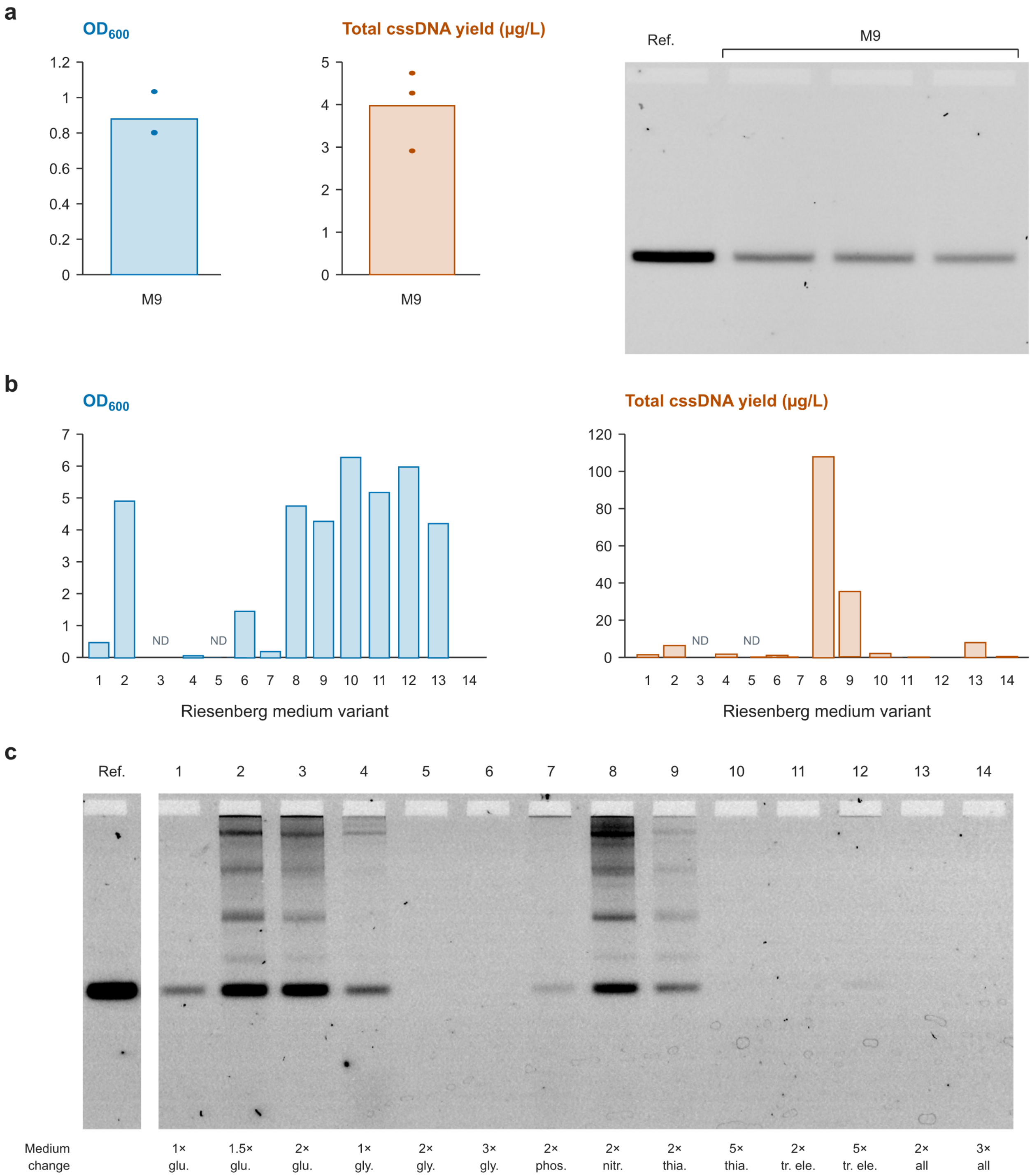

Figure S1. eScaf growth and cssDNA production in M9 and Riesenberg media. (a) Growth and total cssDNA yield of the original eScaf strain in M9 medium. Mean OD600 was 0.88 ± 0.13, and total cssDNA yield was 4.0 ± 1.0 µg/L (mean ± SD; n = 3 biological replicates). (b) Endpoint cell density and total cssDNA yield of the original eScaf strain grown in Riesenberg medium and its variants (n = 1 per condition). (c) Gel analysis of the resulting cssDNA products. The original Riesenberg formulation produced fewer off-target species than the tested variants, with a total cssDNA yield of approximately 1.4 µg/L. Ref.: 3031-nt cssDNA reference.

Riesenberg media composition

Variant	KH_2PO_4 (g/L)	$(NH_4)_2PO_4$ (g/L)	Glucose (g/L)	Glycerol (mL/L)	EDTA (mg/L)	Thiamine (mg/L)	Citric acid (g/L)	$MgSO_4$ (g/L)	Trace elements
1	13.3	4	20	0	8	4.5	1.7	1.2	1x
2	13.3	4	30	0	8	4.5	1.7	1.2	1x
3	13.3	4	40	0	8	4.5	1.7	1.2	1x
4	13.3	4	0	4	8	4.5	1.7	1.2	1x
5	13.3	4	0	8	8	4.5	1.7	1.2	1x
6	13.3	4	0	12	8	4.5	1.7	1.2	1x
7	26.6	4	20	0	8	4.5	1.7	1.2	1x
8	13.3	8	20	0	8	4.5	1.7	1.2	1x
9	13.3	4	20	0	8	9	1.7	1.2	1x
10	13.3	4	0	12	8	22.5	1.7	1.2	1x
11	13.3	4	0	12	8	4.5	1.7	1.2	2x
12	13.3	4	0	12	8	4.5	1.7	1.2	5x
13	26.6	8	40	0	16	9	3.4	2.4	2x
14	39.9	12	60	0	24	13.5	5.1	3.6	3x

Trace element composition

Component	Concentration (mg/L)
CoCl2 · 6H2O	4
MnCl2 · 4H2O	23.5
CuCl2 · 4H2O	1.5
H3BO3	3
Na2MoO4 · 2H2O	2.5
Zn(CH3COO)2 · 2H2O	13
Fe(II) citrate	100

Supplementary Table 1. Riesenberg media recipes. Riesenberg media #1 replicates the batch media recipe listed in DeLisa *et al*. 1999. Trace elements were added together from a single stock solution thus concentrations of all trace element components were increased uniformly. Individual trace element concentrations are listed relative to the entire media.
